# Macrophage polarization modulates neutrophil extracellular trap dynamics and reciprocal macrophage responses

**DOI:** 10.3389/fimmu.2026.1850388

**Published:** 2026-09-01

**Authors:** Laura Blum, Kira Jäger, Makenzie Jones, Julia Elrod, Michael Boettcher, Jasmin Knopf

**Affiliations:** 1 Department of Pediatric Surgery, University Medical Center Mannheim, Heidelberg University, Mannheim, Germany; 2 European Center for Angioscience, Medical Faculty Mannheim, Heidelberg University, Mannheim, Germany; 3 Mannheim Institute for Innate Immunoscience, Medical Faculty Mannheim, Heidelberg University, Mannheim, Germany

**Keywords:** immune cell interaction, macrophages, macrophage plasticity, neutrophils, neutrophil extracellular traps

## Abstract

The interplay between neutrophils and macrophages is central to inflammatory regulation, yet how macrophage polarization governs the homeostasis of neutrophil extracellular traps (NETs) remains poorly understood. Here, we show that the macrophage polarizing environment is a critical determinant of NETs dynamics in human primary cell co-cultures. Using immunofluorescence, flow cytometry, kinetic extracellular DNA assays, and multiplex cytokine profiling, we demonstrate that IFN-γ/LPS (M1-polarized) conditions promote robust but transient NETs formation, accompanied by increased loss of neutrophil membrane integrity and amplified proinflammatory cytokine release. In contrast, IL-4/IL-13 (M2-polarized) conditions restrict the accumulation of NETs-associated extracellular DNA, shift neutrophil death toward an apoptotic rather than a membrane-compromised (lytic) profile, and maintain an attenuated inflammatory profile. Importantly, neutrophil co-culture reciprocally shifts macrophages toward M2-like phenotypes, an effect most pronounced in concurrently polarized (CP) macrophages and diminished once polarization has stabilized. These findings establish the polarizing environment as a bidirectional regulator of NETs homeostasis and neutrophil fate, with implications for understanding the balance between inflammatory amplification and resolution in neutrophil-rich microenvironments.

## 1 Introduction

Neutrophils and macrophages are the principal effector cells of innate immunity and maintain constant bidirectional communication that shapes the onset, progression, and resolution of inflammation (1, 2). Neutrophils are rapidly recruited to sites of tissue damage or infection, where they deploy phagocytosis, degranulation, and the release of neutrophil extracellular traps (NETs) to contain pathogens (3, 4). NETs are web-like structures of decondensed chromatin decorated with histones, myeloperoxidase (MPO), and neutrophil elastase (NE). The formation of NETs is often accompanied by the activation of peptidyl-arginine deiminase 4 (PAD4), which mediates the citrullination of histones, including histone H3, thereby promoting chromatin decondensation and the release of extracellular chromatin. Citrullinated histone H3 (H3Cit) is therefore frequently used as a marker for NET-associated chromatin remodeling, although both PAD4-dependent and PAD4-independent mechanisms of NET formation have been described (5, 6). They are effective antimicrobial effectors but their excessive accumulation or impaired clearance drives tissue injury in sepsis, autoimmunity, and cancer (4, 7, 8).

Macrophages contribute to NETs clearance through extracellular DNase-mediated degradation followed by phagocytic uptake of residual chromatin and associated proteins (9–11). A defining feature of macrophages is their phenotypic plasticity: depending on microenvironmental cues, they adopt activation states ranging from classically activated proinflammatory (M1-like) phenotypes to alternatively activated resolution-promoting (M2-like) phenotypes (12). Although the M1/M2 framework is a simplification of a continuous activation spectrum, it provides a useful conceptual scaffold. Polarization is reversible: if macrophages fail to transition from proinflammatory to pro-resolving states, chronic inflammation can ensue (13–15).

**Figure 1:**
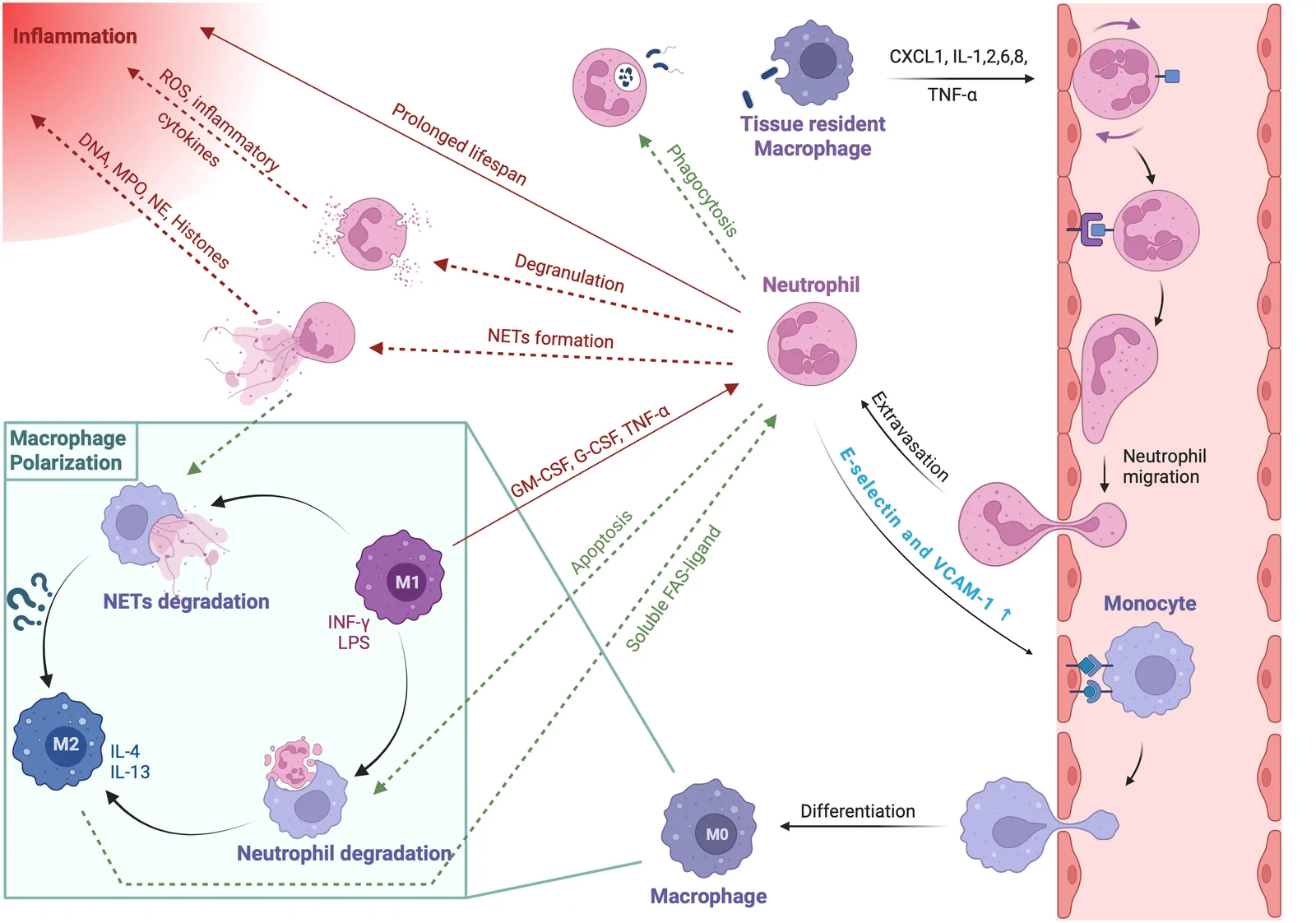
Macrophage–neutrophil interaction in innate immune regulation. Conceptual overview of macrophage–neutrophil crosstalk. Macrophages recognize pathogens and release chemoattractants that promote neutrophil recruitment to the site of infection. Neutrophils respond through degranulation, Neutrophil Extracellular Traps (NETs) formation, and phagocytosis, and recruit additional neutrophils and monocytes, which differentiate into macrophages at the site of infection. Under physiological conditions, neutrophils undergo apoptosis after pathogen clearance and are phagocytosed by macrophages, which thereby acquire an anti-inflammatory phenotype. Persistent neutrophil activation leads to tissue damage through excessive inflammatory responses. The bidirectional influence of macrophage polarization on NETs dynamics and the reciprocal effect of neutrophils on macrophage phenotype are the focus of this study. Green arrows: normal immune response. Red arrows: inflammation or immune overactivation. Abbreviations: NETs, neutrophil extracellular traps; IL, interleukin; TNF-α, tumor necrosis factor-α; IFN-γ, interferon-γ; GM-CSF, granulocyte-macrophages colony-stimulating factor; G-CSF, granulocyte colony-stimulating factor; TGF-β, transforming growth factor-β; CXCL1, C-X-C motif chemokine ligand 1; CX3CR1, C-X3-C motif chemokine receptor 1; LPS, lipopolysaccharide. Created with BioRender.com.

Neutrophil–macrophage crosstalk operates in both directions (**Figure 1**). Tissue-resident macrophages recruit neutrophils through chemokines such as CXCL1, IL-8, and TNF-α (16, 17). Recruited neutrophils, in turn, amplify monocyte influx and can promote M1 polarization of newly differentiated macrophages (18). Resolution begins when macrophages induce neutrophil apoptosis and clear apoptotic cells by efferocytosis, a process that promotes anti-inflammatory reprogramming and M2 polarization (19). Nakazawa et al. reported that M1 macrophages release extracellular DNA in response to NETs, whereas M2 macrophages increase proinflammatory cytokine secretion upon contact with NETotic neutrophils (20), and Haider et al. demonstrated polarization-dependent differences in NETs degradation efficiency (11). However, these studies examined isolated aspects of the macrophage–NETs axis and did not systematically integrate NETs formation, degradation, neutrophil survival pathways, and inflammatory cytokine output within a single experimental framework, nor did they assess how the previous and continuous exposure of macrophages to polarizing stimuli influence their response to neutrophil co-culture.

What remains unclear is how the full spectrum of macrophage activation states coordinately regulates NETs formation, degradation, neutrophil survival, and inflammatory cytokine output, and how neutrophils reciprocally shape macrophage polarization during this process (21). Here, we systematically address this question using an autologous human primary cell system. We demonstrate that the macrophage polarizing environment shapes NETs dynamics through coordinated regulation of NETs induction, NETs-associated extracellular DNA dynamics, and neutrophil death pathways, and that neutrophils reciprocally drive macrophages toward M2-like phenotypes, an effect critically dependent on the degree of prior macrophage polarization and the polarizing environment. These findings identify the polarization environment, stimulation history, and macrophage phenotypic plasticity as a central node in the bidirectional regulation of NETs homeostasis.

## 2 Methods

### 2.1 Ethics statement and blood collection

Whole blood was collected from healthy adult volunteers (age range: 20–40 years; both male and female donors included) in EDTA tubes (Sarstedt, Germany) after written informed consent was obtained. The study was approved by the Ethics Committee II of Heidelberg University (approval number: 2023-587) and all procedures were performed in accordance with the principles of the Declaration of Helsinki.

### 2.2 Isolation of PBMCs

Whole blood was collected in EDTA tubes (Sarstedt), pooled in 50 ml tubes and diluted with PBS. Diluted blood was carefully layered onto Histopaque^®^-1077 (Sigma-Aldrich) without mixing and centrifuged for 30 minutes at 460 × g and 20 °C without brake. The plasma layer was removed, and the layer of peripheral blood mononuclear cells (PBMCs) was collected. PBMCs were washed with PBS. The cells were counted with Acridine Orange/Propidium Iodide (PI) using a Luna-FL™ cell counter (Logos Biosystems).

### 2.3 Isolation of monocytes by magnetic separation with CD14 beads

Cells were resuspended in MACS buffer (0.5% BSA, 2 mM EDTA) and mixed with CD14 microbeads (Miltenyi) and incubated at 4 °C for 15 minutes. MACS buffer was added, gently mixed, and the cells were centrifuged at 300 × g for 6 minutes. CD14 + monocytes were isolated via LS column (Miltenyi) and a QuadroMACS™ magnetic separator (Miltenyi). The cell suspension was added to the LS column and washed three times with MACS buffer. CD14+ cells were eluted and seeded as follows.

### 2.4 Seeding and differentiation of monocytes

CD14^+^ monocytes were counted and seeded at a concentration of 2.5 × 10^5^ cells/well in 1 ml (24-well plate) or at a concentration of 1.0 × 10^5^ cells/well in 200 µl (48-well plates). The cells were seeded in RPMI-1640 (Gibco) supplemented with 2% autologous human serum and 100 ng/ml human M-CSF (Immunotools) (Mreg medium). The cultures were maintained for 6 days at 37 °C and 5% CO_2_ without medium changes.

### 2.5 Polarization of macrophages

On day 6 the macrophages were polarized to M1 (IFN-γ/LPS) or M2 (IL-4/IL-13) phenotypes. For M1, the medium contained 100 ng/ml M-CSF (11343115), 20 ng/ml recombinant human IFN-γ (both Immunotools, 11343536) and 100 ng/ml LPS (Kleb. pneumoniae; Sigma, L4268). For M2, the medium contained 100 ng/ml M-CSF, 20 ng/ml recombinant human IL-4 and recombinant human IL-13 (both 20 ng/ml) (all Immunotools, 11340043, 11340133). M0 controls received only 100 ng/ml M-CSF (Immunotools). The cells were incubated for 24–48 hours at 37 °C and 5% CO_2_. Throughout this study, “concurrently polarized” refers to macrophages that received polarizing stimuli (IFN-γ/LPS or IL-4/IL-13) simultaneously with neutrophil addition on day 7, whereas “pre-polarized” refers to macrophages that were polarized for 24 h prior to neutrophil co-culture and 24 h together with neutrophils (48 h polarization stimuli exposure).

### 2.6 Isolation of neutrophils (PMNs)

Polymorphonuclear cells (PMNs) were collected together with PBMCs. Erythrocytes were lysed with ice-cold ddH_2_O, followed by the addition of ice-cold 10× PBS (Gibco). Erythrocyte lysis was repeated one more time. Freshly isolated neutrophils were co-cultured in a 2:1 ratio (2x10^5^: 1x10^5)^ with monocytes/macrophages for 24 h.

### 2.7 Cell labeling

Approximately 10^6^ cells were labeled with CellTrace™ CFSE (Abcam, ab113853) or CellTrace™ Far Red (Thermo Fisher Scientific, C34564) according to the manufacturer’s instructions. For CFSE labeling, cells were incubated with 5 µM CellTrace™ CFSE for 20 minutes at 37 °C in the dark, washed with RPMI-1640, incubated briefly at room temperature, and then centrifuged. For Far Red labeling, cells were incubated with 1 µM CellTrace™ Far Red for 15 minutes at 37 °C in the dark, diluted with RPMI-1640, incubated at room temperature, and then centrifuged.

### 2.8 Flow cytometry

#### 2.8.1 7-color polarization panel

Cells were detached with 10 mM EDTA (Gibco) in DPBS (Gibco). Wells were scraped with a cell scraper and washed with DPBS. Cells were fixed with 4% PFA (VWR) for 20 min and permeabilized for 10 min with 0.2% Triton X-100 (Sigma) in blocking buffer (10% human serum, 4 µL/sample Fc-block (BioLegend), 5 µL/sample NovaRed block (Thermo Fisher Scientific)). Cells were blocked for 30 min, and then fluorophore-conjugated antibodies were added. A seven-color panel was created for polarization analysis: Brilliant Violet 605™ CD14 (BioLegend, 301834), CD68-PE (Thermo Fisher Scientific, 12-0689-42), NovaRed CD86 (Thermo Fisher Scientific, H021T03R06-A), PE/Cyanine7 CD163 (BioLegend, 333614), and Pacific Blue CD16 (BD, 558122). Macrophages were previously labeled with CellTrace™ CFSE, and neutrophils with CellTrace™ Far Red. The gating strategy is displayed in **Supplementary Figure 1**.

### 2.9 Annexin/PI staining

Collected cells were resuspended in Annexin binding buffer (2.5 mM CaCl2, pH 7.5). Annexin-FITC (ImmunoTools, 31490013X2) (1:100) was added and incubated for 30 min and afterwards 0.5 µl Propidium Iodide (PI) (BioLegend) was added for another 15 minutes.

### 2.10 Hoechst/Annexin V/PI staining

Cells were collected and resuspended in Ringer solution. Hoechst 33342 (Thermo Fisher Scientific, 1:1000), Annexin V-FITC (ImmunoTools, 1:100), and Propidium Iodide (PI, 0.5 µl per sample) (BioLegend) were added. Samples were analyzed immediately by flow cytometry. The gating strategy is displayed in **Supplementary Figure 2**.

### 2.11 MPO-Sytox™ orange staining

Cells were collected and stained with MPO-eFluor 450 (Thermo Fisher Scientific, 48-1299-42) 1:200 in FACS buffer and Sytox™ Orange (Thermo Fisher Scientific, S34861) (1:1000). The gating strategy is displayed in **Supplementary Figure 3**.

### 2.12 H3cit-MPO-Sytox™ orange staining

Macrophages were co-cultured with CFSE-labeled neutrophils at a 1:2 ratio in 48-well plates for 24 h, as described above. Following incubation, cells were collected and transferred to 5 ml polystyrene tubes as described above. Cells were stained with primary anti-citrullinated histone H3 antibody (H3cit; Abcam, ab5103) at a dilution of 1:300. After washing cells were stained with Alexa Fluor™ 700-conjugated secondary antibody (Thermo Fisher Scientific, A-21038; 1:300), MPO-eFluor™ 450 antibody (Thermo Fisher Scientific; 1:100), and Sytox™ Orange (Thermo Fisher Scientific; 1:1000). The gating strategy is displayed in **Supplementary Figure 4**.

### 2.13 Transwell co-culture

Macrophages were generated and cultured in 24-well plates as described above. On day 7, neutrophils were seeded into 0.4 µm pore-size Transwell inserts (Corning) and placed into the wells containing macrophages. Co-cultures were maintained for 24 h at 37 °C and 5% CO_2_. After incubation, macrophages were detached with 10 mM EDTA in DPBS, collected by scraping, and stained for polarization analysis as described above. Neutrophils were collected from the Transwell inserts and stained for NETs formation using MPO-eFluor 450 and Sytox™ Orange.

### 2.14 LEGENDplex™ immune panel

LEGENDplex™ Human Inflammation Panel (13-plex, BioLegend) was used to analyze samples. The assay was performed as specified by the manufacturer.

Data were analyzed using FlowJo 10.10.1. Provided standards were used to calculate concentrations in pg/ml.

### 2.15 NE activity assay

Fresh neutrophils were co-cultured 2:1 with seeded macrophages. Cytokines for M1 and M2 cultures were diluted in RPMI-1640 for stimulation. The fluorogenic NE substrate MeOSuc-AAPV-AMC (Santa Cruz Biotechnology) was added to the medium at a final concentration of 100 µM. The substrate-containing medium was added, immediately followed by the addition of the neutrophil suspension. Fluorescence was recorded for 24 hours at 37 °C using a Tecan SparkCyto plate reader (Ex 360 nm, Em 465 nm).

### 2.16 Far Red -Sytox™ green assay

Fresh neutrophils were stained with CellTrace™ Far Red according to the manufacturer’s instructions. Neutrophils were co-cultured 2:1 with macrophages. For stimulation, cytokines for M1 and M2 cultures were diluted in RPMI-1640 and Sytox™ Green (Thermo Fisher Scientific, S7020) was added at a dilution of 1:1200. Sytox™ Green-containing medium was added immediately followed by the neutrophil suspension. Fluorescence was recorded for 24 hours at 37 °C on a Tecan SparkCyto plate reader (Ex 488 nm, Em 535 nm).

### 2.17 PAD4 inhibitor -sytox™ green assay

Freshly isolated neutrophils were co-cultured with macrophages at a neutrophil-to-macrophage ratio of 2:1. For stimulation, M1- and M2-polarizing cytokines were diluted in RPMI-1640 (Thermo Fisher Scientific) medium. SYTOX™ Green (Thermo Fisher Scientific) was added at a final dilution of 1:1200 immediately before adding the neutrophil suspension. Where indicated, the PAD4 inhibitor GSK-484 (Selleckchem, S7803, stock concentration: 1 mM) was added at a final concentration of 10 µM. Fluorescence signals were recorded over 24 h at 37 °C using a Tecan SparkCyto plate reader (SYTOX™ Green: Ex 488 nm, Em 535 nm).

### 2.18 NETs ELISAs

96-well plates were coated with anti-neutrophils elastase (R&D, MA391673) or anti-myeloperoxidase (Merck, 07-496-I) in coating buffer (0.1 M Na2CO3, 0.1M NaHCO3; pH 9.6) overnight at 4 °C. Plates were washed with PBS-T and blocked with blocking buffer (3% BSA in PBS) for 2 hours. Subsequently, samples were added and incubated for 2 hours. Samples were incubated with peroxidase-conjugated anti-DNA antibody (1:40 in incubation buffer, Cell death detection kit, Roche) for 90 minutes. TMB substrate (BioLegend) was added and developed for 30 minutes before stopping the reaction with 25% H_2_SO_4_. Absorbance was measured at 450 nm with 620 nm as reference using a Tecan SparkCyto plate reader. For normalization, the mean absorbance of blank wells was subtracted from all sample wells.

### 2.19 Immunofluorescence staining

Cells were seeded and incubated on coverslips. Cells were washed with PBS and fixed with 4% paraformaldehyde (VWR), washed, and permeabilized with PBS + 0.1% Triton X-100 (Sigma). Cells were blocked in 10% goat serum (Biowest SAS/VWR). Cells were incubated overnight in blocking buffer with primary antibodies. For polarization: CD68 (clone D4B9C, Cell Signaling Technology, 26042SF, 1:200), CD80 (clone 1E2F10, Proteintech, 66406-1-Ig, 1:50), CD206 (clone 2A6A10, Proteintech, 60143-1-Ig, 1:200), For NETs: Myeloperoxidase (MPO; rabbit polyclonal, Abcam, ab9535, 1:150), double-stranded DNA (dsDNA; clone 35I9 DNA, Abcam, ab27156, 1:500), neutrophil elastase (NE; clone NP57, Abcam, ab254178, 1:100), and citrullinated histone H3 (H3Cit; rabbit polyclonal, Abcam, ab5103, 1:100). The next day, cells were washed and incubated with labeled secondary antibodies in blocking buffer. For polarization: Goat anti-rabbit IgG AF488 1:500 (Invitrogen, A11008), goat anti-mouse IgG Cyanine5 (Invitrogen, A10524) 1:500. For NETs: Goat anti-mouse IgG AF568 2 µg/ml (Invitrogen, A11004), goat anti-rabbit IgG AF Plus 647 (Invitrogen, A32733)1:500, and SYTOX™ Green 1:1000. Cells were stained with DAPI (Sigma, 1:1000) solution and mounted with fluorescence mounting medium (Dako/Agilent) on slides (Epredia). Images were acquired using a Keyence BZ-X810 (20x, 60x magnification).

### 2.20 Live cell imaging

Live-cell imaging was performed using a Leica Mica micro hub widefield microscope with a 20× objective. Cells were imaged for 14 h at 37 °C and 5% CO_2_ with images acquired every 15 min. Neutrophils, macrophages, and macrophage–neutrophil co-cultures were cultured in phenol red-free RPMI (Gibco) medium supplemented with M-CSF and the respective cytokines. SYTOX ™ Green was added at a dilution of 1:1000 to monitor extracellular DNA. Far-red labeling was used to monitor neutrophils. Brightfield, GFP (SYTOX Green), and Cy5 (Far-Red) channels were acquired over time.

### 2.21 Statistical analysis

Statistical analyses were performed using GraphPad Prism v10.2.3. Considering the limited sample size and inter-donor variability, non-parametric tests were applied. Comparisons between two independent groups were performed using the Mann–Whitney U test. For comparisons of multiple groups, including concurrently polarized (CP) and pre-polarized (PP) macrophages conditions, a Kruskal–Wallis test followed by Dunn’s multiple comparisons test was used.

Individual biological replicates are shown for all experiments. Data are presented as mean ± standard deviation (SD) to illustrate donor-to-donor variability. The number of independent donors is indicated in each figure legend (n = 5 or n = 10). Exact p-values are reported in the figure panels, and p < 0.05 was considered statistically significant.

## 3 Results

### 3.1 Monocyte-to-macrophage differentiation reveals progressive phenotypic maturation with incomplete polarization

**Figure 2:**
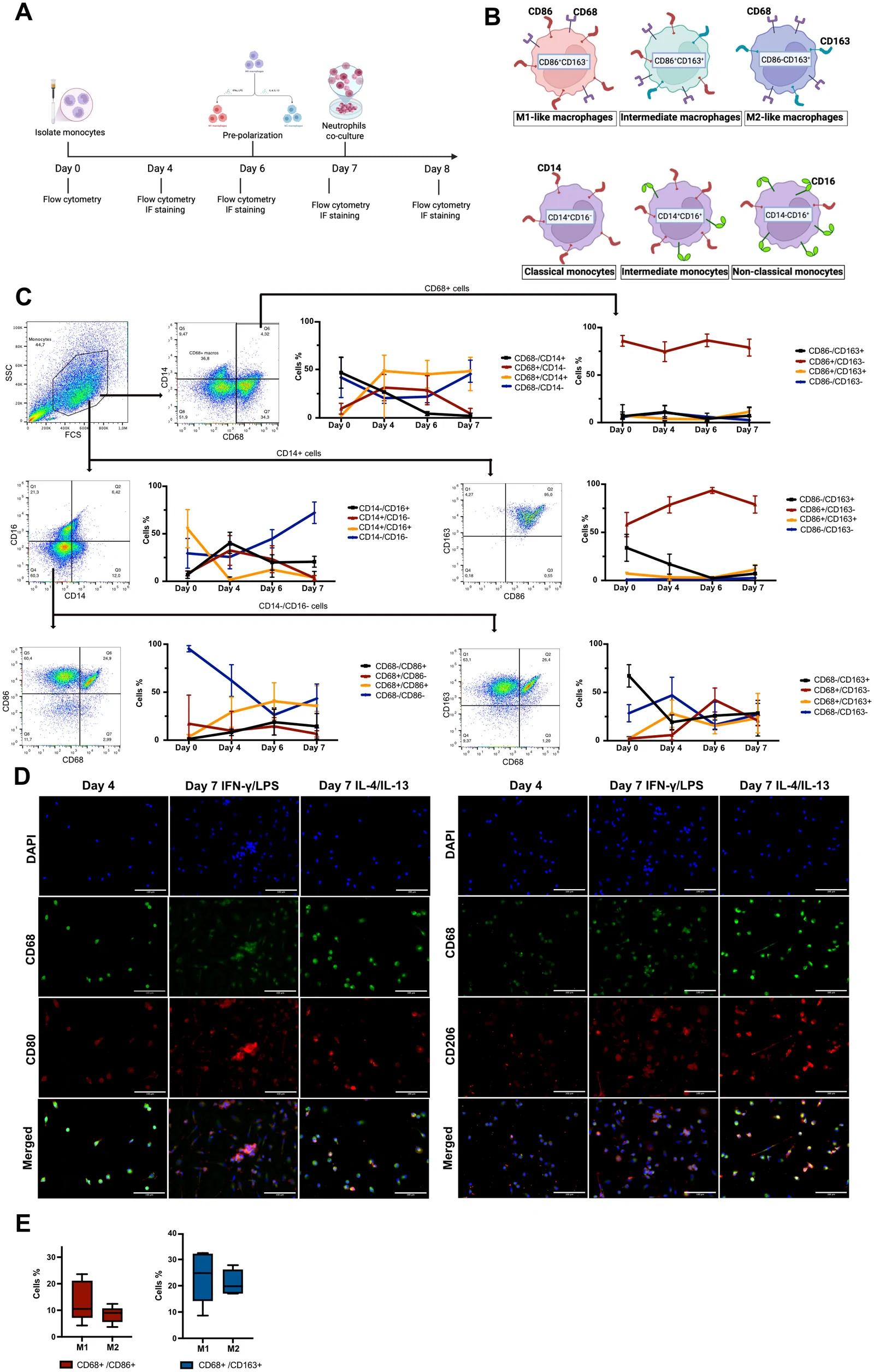
Time-dependent differentiation and phenotypic maturation of human monocytes into macrophages. **(A)** Schematic of the experimental timeline. Upper panel: culture conditions; lower panel: analyses at each time point. Analyses on days 6 and 7 were conducted prior to polarization. **(B)** Classification of monocyte subsets (CD14/CD16) and macrophages phenotypes (CD68/CD86/CD163). **(C)** Flow cytometric analysis of monocyte subsets at days 0, 4, 6, and 7 without polarization or neutrophils. Dot plots show CD14/CD16, CD14^+^CD86/CD163, CD86/CD163, and CD14/CD68. **(D)** Representative immunofluorescence images on days 4 and 7 (pre-polarization) under IFN-γ/LPS and IL-4/IL-13 conditions; DAPI (blue), CD68 (green), CD80 or CD206 (red). Scale bars, 100 µm. **(E)** Quantification of CD68^+^CD86^+^ and CD68^+^CD163^+^ macrophages under IFN-γ/LPS and IL-4/IL-13 conditions on day 7 (pre-polarization). n = 5 independent donors.

To characterize the phenotypic trajectory of monocyte-to-macrophage differentiation, freshly isolated human CD14^+^ monocytes were cultured with M-CSF for 7 days, and surface marker expression was assessed longitudinally by flow cytometry and immunofluorescence (**Figure 2A**). Human monocytes were classified into classical (CD14^+^CD16^–^), intermediate (CD14^+^CD16^+^), and non-classical (CD14^–^CD16^+^) subsets based on CD14/CD16 co-expression. Macrophages were further categorized as M1-like, M2-like, or intermediate depending on CD68, CD86, and CD163 expression (**Figure 2B**).

Flow cytometry revealed a time-dependent shift in monocyte subset composition (**Figure 2C**). At day 0, intermediate CD14^+^CD16^+^ monocytes predominated with smaller classical and non-classical fractions. Over time, CD14 expression declined progressively while CD16 expression increased, and CD14^–^CD16^+^ cells became more prominent by days 6–7 consistent with ongoing maturation. In parallel, the CD68^+^CD14^–^ population expanded with a transient CD68^+^CD14^+^ intermediate on day 4, whereas CD68^–^CD14^+^ cells decreased confirming differentiation into macrophages. Immunofluorescence stainings on days 4 and 7 corroborated these findings, revealing progressive morphological changes alongside the emergence of polarization markers (**Figure 2D**). Both CD80 and CD206 were detectable at low levels in all cultures, with their simultaneous presence indicating incomplete phenotypic separation at these time points. Importantly, CD80/CD206 expression was predominantly observed in the CD68^+^ compartment suggesting that polarization-associated features became most apparent after the acquisition of macrophage identity.

Analysis of the macrophage compartment (CD68^+^ cells) for CD86 and CD163 did not yield clearly separable M1-like versus M2-like populations over the culture period. Instead, CD163 expression increased progressively while CD86 remained low suggesting a tendency toward a partially M2-like mixed phenotype, consistent with the known M2-skewing effect of M-CSF-driven differentiation (22). After polarization on day 7 (**Figure 2E**), both M1 and M2 cultures contained mixed populations: IFN-γ/LPS conditions enriched CD86^+^ cells relative to IL-4/IL-13 conditions but substantial M2-like fractions persisted, and M2 cultures likewise harbored both phenotypes. Taken together, these data indicate marked phenotypic heterogeneity *in vitro* and incomplete separation of marker-defined macrophage populations under the applied polarization conditions.

### 3.2 Neutrophils promote CD163-associated (M2-like) macrophage polarization in a plasticity-dependent manner

**Figure 3:**
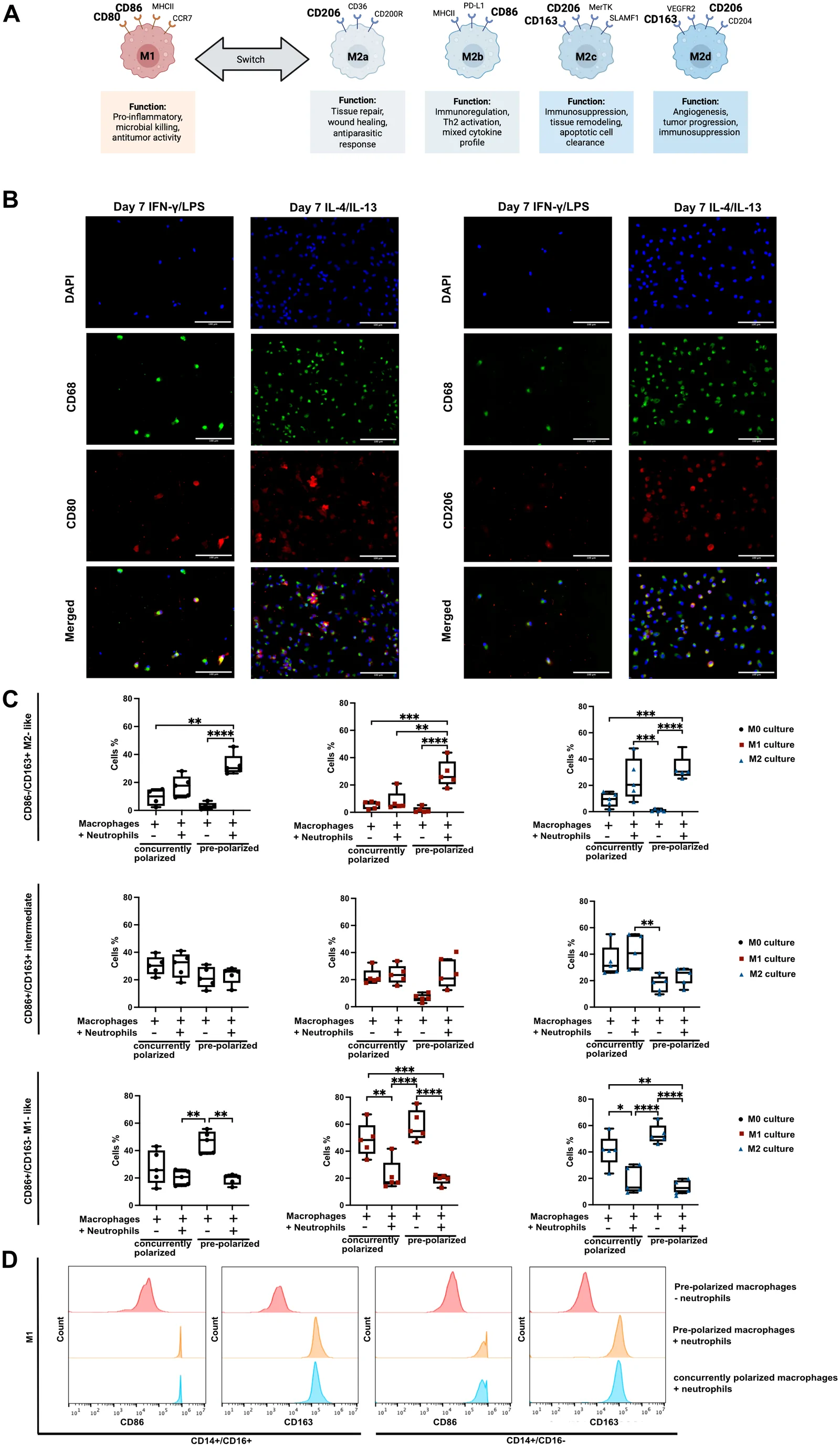
Neutrophil-dependent modulation of macrophage polarization states. **(A)** Schematic of macrophage polarization states and surface marker profiles. M2 subsets (M2a–d) are defined by differential expression of CD163, CD206, and CD86. **(B)** Representative immunofluorescence images under IFN-γ/LPS and IL-4/IL-13 conditions after 24 h neutrophil co-culture; DAPI (blue), CD68 (green), CD80 (red). Scale bars, 100 µm. **(C)** Quantification of macrophage subsets (CD86^–^CD163^+^ [M2-like], CD86^+^CD163^+^ [intermediate], CD86^+^CD163^–^ [M1-like]) following co-culture with neutrophils under concurrently polarized and pre-polarized conditions. **(D)** Representative flow cytometry histograms of CD86 and CD163 mean fluorescence intensity (MFI) in CD14^+^CD16^+^ and CD14^+^CD16^–^ macrophage subsets. *p < 0.05, **p < 0.01, ***p < 0.001, ****p < 0.0001 (Kruskal–Wallis test followed by Dunn’s multiple comparisons test). n = 5 independent donors.

To determine whether neutrophils modulate macrophage polarization, autologous neutrophils were added to macrophage cultures on day 7 and the polarization profile was assessed after 24 h by flow cytometry and immunofluorescence. **Figure 3A** illustrates the spectrum of macrophage activation states and highlights selected surface markers associated with classical inflammatory (M1-like) and alternative/regulatory (M2-like) macrophage phenotypes. Since macrophage activation represents a continuum rather than fixed subsets, marker combinations including CD86, CD163 and CD206 were used to characterize polarization-associated changes.

Immunofluorescence staining for CD68, CD80, and CD206 under M1 and M2 polarization conditions after 24 h of neutrophil co-culture confirmed the presence of both M1- and M2-associated markers under all conditions supporting the presence of heterogeneous macrophage populations rather than fully separated activation states (**Figure 3B**).

Quantification of macrophage subsets revealed that neutrophil co-culture consistently shifted macrophage phenotypes toward CD163-associated (M2-like) profiles across all stimulation conditions (**Figure 3C**). This neutrophil-associated accumulation of CD163-dominant macrophage phenotypes was more pronounced under conditions of concurrent polarization than under conditions of pre- polarization, suggesting that the timing and length of exposure to polarization stimuli influence macrophage responses during neutrophil co-culture. Conversely, M1-like macrophages were reduced in neutrophil co-cultures, particularly under pre-polarized conditions, consistent with a shift from CD86- to CD163-dominant phenotypes. Intermediate macrophage populations showed comparatively minor changes.

Analysis of marker expression intensity in monocyte-derived subpopulations revealed that neutrophils differentially modulated CD86 and CD163 expression depending on the monocyte subset of origin (**Figure 3D**). In macrophages derived from CD14^+^CD16^+^ monocytes, neutrophil co-culture was associated with altered CD86 and CD163 intensities suggesting active modulation of polarization-associated markers. In contrast, CD14^+^CD16^–^-derived macrophages showed a distinct modulation pattern indicating that neutrophil-driven phenotypic shifts depend on the monocyte subset of origin. Notably, polarization stimuli alone, without neutrophils, had only limited effects on CD86 and CD163 expression underscoring the potency of neutrophil-derived signals. The persistent co-expression of CD86 in M2 cultures may reflect an M2b-like activation state characterized by retention of costimulatory molecules alongside regulatory functions rather than incomplete polarization.

### 3.3 Macrophages polarization environment determines NETs formation dynamics

**Figure 4:**
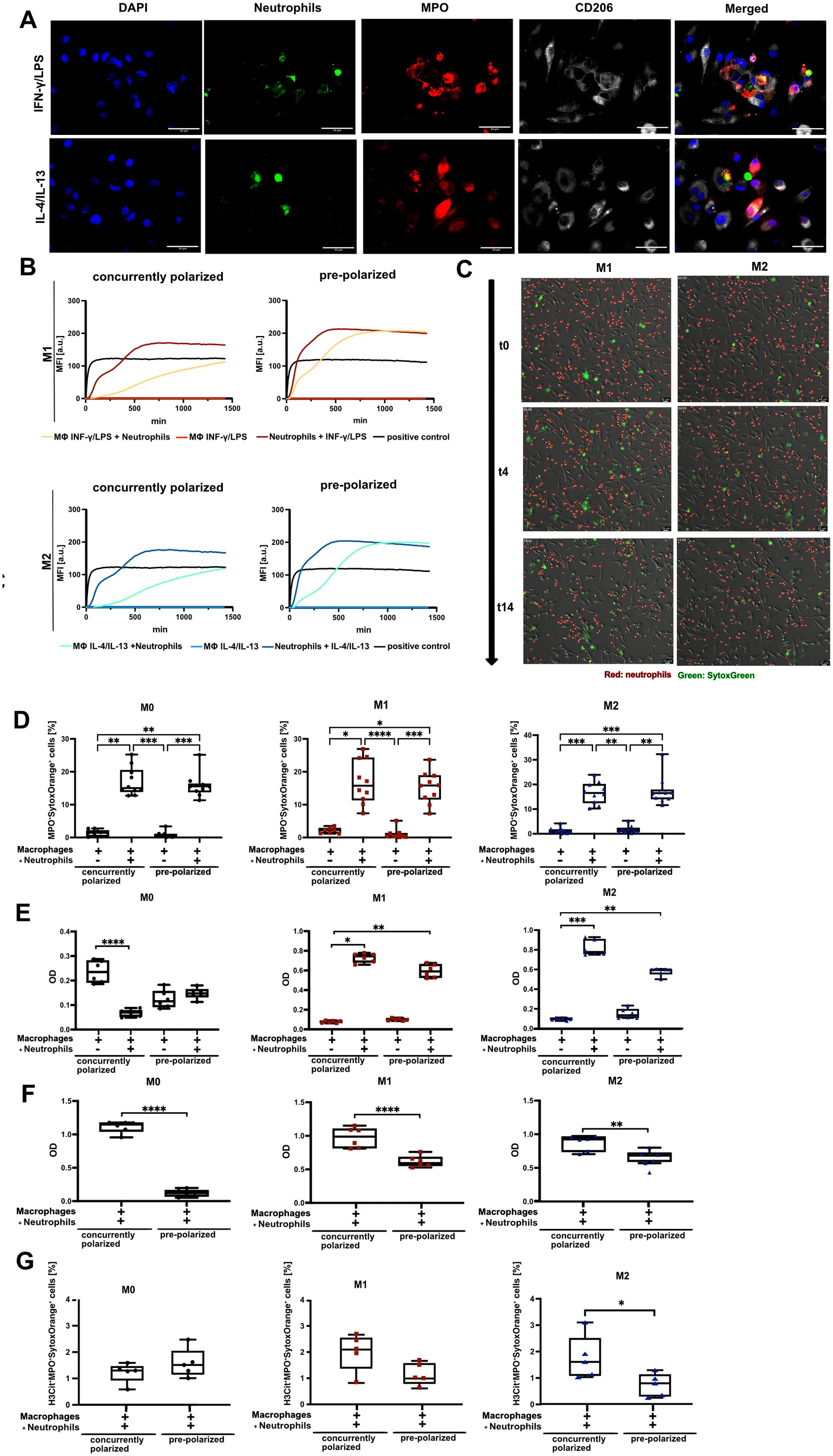
NETs formation in macrophage–neutrophil co-cultures is modulated by macrophage polarization state and environment. **(A)** Representative immunofluorescence images of M1 and M2 co-cultures; DAPI (blue), neutrophils (green), MPO (red), CD206 (grey). Scale bar, 50 µm. **(B)** Kinetic NE activity in concurrently polarized and pre-polarized co-cultures. Mean fluorescence intensity (MFI) over time after IFN-γ/LPS or IL-4/IL-13 stimulation, ± neutrophils. **(C)** Representative live-cell imaging images of concurrently polarized M1/M2 macrophage co-culture. Neutrophils (red), cell-free DNA (green), Scale bar, 20 µm at t0, t4 and t14 **(h)**. Leica Mica, 20x magnification, 14h run, imaging: every 15 min at 37 °C, 5% CO_2_. **(D)** NETs quantification by flow cytometry (MPO^+^–Sytox™ Orange^+^ events) in M0, M1, and M2 co-cultures. **(E)** MPO–DNA ELISA. **(F)** NE–DNA ELISA. **(G)** Quantification of H3cit^+^MPO^+^Sytox Orange^+^ events in concurrently polarized (CP) and pre-polarized (PP) M0, M1, and M2 macrophage–neutrophil co-cultures by flow cytometry. *p < 0.05, **p < 0.01, ***p < 0.001, ****p < 0.0001 (Mann–Whitney U test for comparisons between two groups and a Kruskal–Wallis test followed by Dunn’s multiple comparisons test for comparisons of more than two groups). **(A, B, E–G)** n = 5 independent donors, **(C)** n = 1 donor, **(D)** n = 10 independent donors.

To assess NET-associated responses under distinct polarizing environments, neutrophils were co-cultured with M0 macrophages or under IFN-γ/LPS (M1-polarized) and IL-4/IL-13 (M2-polarized) conditions. Immunofluorescence staining revealed polarization-dependent patterns of neutrophil–macrophage interactions (**Figure 4A**). In M1 (IFN-γ/LPS) co-cultures, increased MPO-positive extracellular signals were observed in association with neutrophils and macrophages, whereas M2 (IL-4/IL-13) co-cultures showed a more restricted MPO distribution, with signals frequently located near CD206^+^ macrophages. These observations suggest differences in the accumulation and spatial organization of NET-associated components depending on macrophage polarization state. Additional representative DNA-focused images illustrating the overall distribution and morphology of NET-associated extracellular DNA structures are provided in **Supplementary Figure 5**. These images are intended for qualitative illustration only and are not used for quantitative comparison between experimental conditions or as definitive visualization of classical filamentous NET structures.

Kinetic analysis of extracellular DNA release demonstrated that macrophage polarization influenced the dynamics of neutrophil-derived signals (**Figure 4B**). M1-associated co-cultures showed sustained extracellular DNA accumulation, whereas M2-associated co-cultures displayed delayed and reduced signal progression. Although kinetics differed between concurrent and pre- polarization conditions, the overall pattern indicates that the polarizing environment and stimulation history influence NETs-associated extracellular DNA accumulation and persistence.

Live-cell imaging revealed that the dynamics of extracellular DNA from neutrophils varied depending on macrophage polarization (**Figure 4C**). In a quantitative comparison, co-cultures with IFN-γ/LPS-stimulated (M1-polarized) macrophages showed increased accumulation and formation of SytoxGreen-positive extracellular DNA structures, whereas IL-4/IL-13 (M2-polarized) macrophages exhibited reduced accumulation of extracellular DNA. In addition, IL-4/IL-13-stimulated (M2-polarized) macrophages appeared to degrade DNA more extensively, as the green signal was less pronounced after 14 hours than in the M1 culture, consistent with reduced accumulation, although whether this reflects enhanced degradation, cellular uptake, or reduced further release cannot be determined from this assay. Furthermore, this live-cell imaging suggested that neutrophil-derived material was also processed by macrophages, similar to cell-free DNA, although this was not quantitatively assessed (**Supplementary Videos 1, 2**).

Flow cytometric quantification of MPO^+^SytoxOrange^+^ events further confirmed that NETs-associated signals were strongly influenced by macrophage polarization (**Figure 4D**). Co-culture with macrophages markedly increased MPO^+^SytoxOrange^+^ signals compared with macrophages cultured without neutrophils, with the strongest response observed under M1-polarizing conditions. In contrast, M2-associated environments showed reduced NETs-associated signals after macrophage pre-polarization, supporting a role for macrophage activation state in regulating extracellular DNA accumulation. These findings were further supported by the levels of MPO–DNA and NE–DNA in cell culture supernatants (**Figures 4E, F**), demonstrating reduced NETs-associated DNA–protein complexes after macrophage pre-polarization, particularly in IL-4/IL-13 conditions.

To further strengthen the identification of MPO^+^SytoxOrange^+^ events as NETs, flow cytometric detection of H3Cit^+^MPO^+^SytoxOrange^+^ neutrophil-derived events was performed (**Figure 4G**). Quantification revealed polarization-dependent differences between concurrently polarized and pre-polarized macrophage cultures. Pre-polarization reduced H3Cit^+^MPO^+^SytoxOrange^+^ events, particularly under IL-4/IL-13 (M2-polarized) conditions, indicating decreased accumulation of NETs-associated chromatin structures in the presence of alternatively activated macrophages. Taken together these data indicate altered NETs-associated chromatin/protein complex accumulation in co-culture with differentially polarized macrophages.

### 3.4 IL-4/IL-13-polarizing conditions limit the accumulation of NETs-associated extracellular DNA and shift neutrophil death towards an apoptotic profile

To investigate NETs-associated extracellular DNA accumulation and neutrophil fate, neutrophils were cultured under concurrent or pre-polarization conditions in M0, IFN-γ/LPS (M1-polarized), and IL-4/IL-13 (M2-polarized) cultures. Extracellular DNA dynamics and cell-death-associated profiles were examined using immunofluorescence, real-time extracellular DNA monitoring, PAD4 inhibition, and flow cytometry.

**Figure 5:**
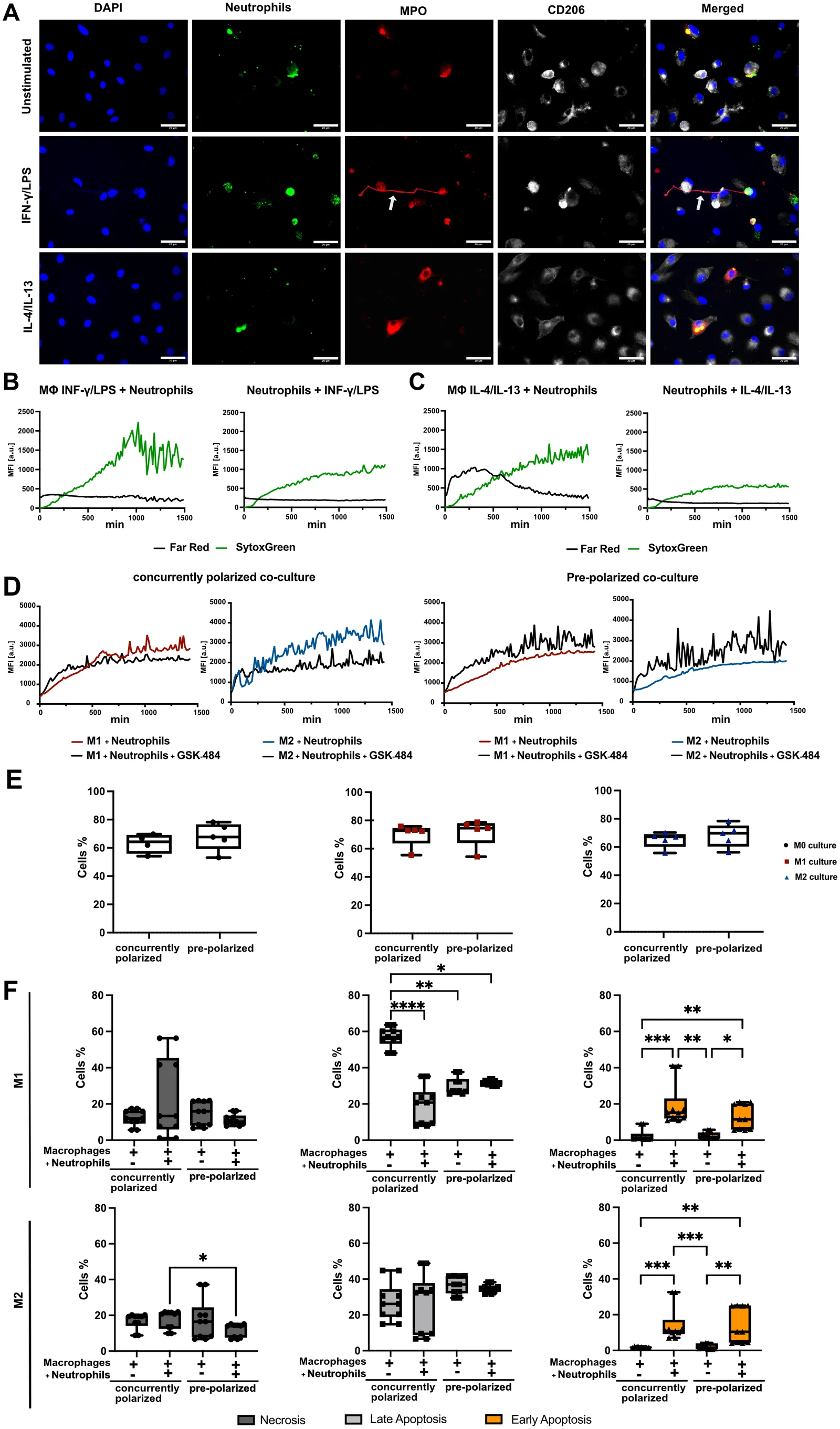
Macrophage-dependent regulation of NETs-associated extracellular DNA dynamics and neutrophil cell death. **(A)** Representative immunofluorescence images of M0, M1, and M2 co-cultures; DAPI (blue), neutrophils (green), MPO (red), CD206 (grey). White arrows: NETs structures. Scale bars, 20 µm. **(B, C)** Real-time NETs-associated extracellular DNA dynamics assay. Sytox™ Green fluorescence (extracellular DNA, green) and CellTrace™ Far Red (neutrophils, black) over time in co-cultures with IFN-γ/LPS- or IL-4/IL-13-polarized macrophages or neutrophils alone. **(D)** Real-time quantification of extracellular DNA dynamics in M0, M1, and M2 macrophage–neutrophil co-cultures treated with or without the PAD4 inhibitor GSK-484. Concurrently polarized and pre-polarized macrophage conditions were analyzed over time by Sytox™ Green mean fluorescence intensity (MFI, a.u.). **(E)** Quantification of viable neutrophils in concurrently polarized (CP) and pre-polarized (PP) M0, M1, and M2 macrophage–neutrophil co-cultures assessed by Annexin V/PI/Hoechst flow cytometry. **(F)** Quantification of neutrophil necrosis, late apoptosis, and early apoptosis by Annexin V/PI flow cytometry under concurrently polarized and pre-polarized IFN-γ/LPS and IL-4/IL-13 conditions. *p < 0.05, **p < 0.01, ***p < 0.001, ****p < 0.0001 (Mann–Whitney U test for comparisons between two groups and Kruskal–Wallis test followed by Dunn’s multiple comparisons test for comparisons of more than two groups). **(A–E)** n = 5 independent donors. **(F)** n = 10 independent donors.

Immunofluorescence analysis revealed MPO-positive structures associated with extracellular DNA, supporting the presence of NETs-associated components (**Figure 5A**). In IFN-γ/LPS-stimulated co-cultures, prominent extracellular MPO-positive structures were observed, whereas IL-4/IL-13-stimulated co-cultures displayed more restricted MPO signals, frequently located near CD206^+^ macrophages. These findings suggest polarization-dependent differences in NETs-associated extracellular DNA accumulation and potential macrophage-mediated processing.

To evaluate NETs-associated extracellular DNA dynamics over time, neutrophils were labeled with CellTrace™ Far Red, and extracellular DNA was detected with Sytox™ Green (**Figure 5B**). In co-cultures with IFN-γ/LPS-stimulated macrophages, Sytox™ Green fluorescence increased rapidly followed by a marked decline at later time points. This pattern is consistent with robust extracellular DNA detection followed by a decline in signal, which may reflect degradation, cellular uptake, or reduced further release. In contrast, IL-4/IL-13-stimulated macrophage co-cultures exhibited reduced Sytox™ Green accumulation and altered kinetic profiles, indicating altered extracellular DNA accumulation and persistence. The distinct kinetic patterns between neutrophils alone and macrophage co-cultures demonstrate differences in extracellular DNA dynamics.

To determine the contribution of PAD4-dependent chromatin decondensation to the observed extracellular DNA signals, co-cultures were treated with the PAD4 inhibitor GSK-484 (**Figure 5D**). PAD4 inhibition altered Sytox™ Green kinetics in both M1 and M2 co-cultures. In concurrently polarized co-cultures, GSK-484 reduced the accumulation of extracellular DNA, supporting a contribution of PAD4-dependent chromatin decondensation to the release of extracellular DNA by neutrophils. In contrast, pre-polarized macrophage environments exhibited persistent or increased extracellular DNA signals despite PAD4 inhibition, suggesting that extracellular DNA accumulation is not exclusively driven by PAD4-dependent NETs formation. Instead, these results suggest additional contributions from PAD4-independent neutrophil responses, alternative cell death pathways, and macrophage-dependent regulation of the persistence and clearance of extracellular DNA.

To exclude that reduced extracellular DNA signals resulted from lytic cell death of neutrophils, Hoechst-positive live neutrophil populations were quantified after co-culture (**Figure 5E**). Comparable frequencies of Hoechst^+^ neutrophils between concurrently polarized and pre-polarized macrophage cultures demonstrated preservation of neutrophil populations throughout the experiment. These findings indicate that differences in NETs-associated extracellular DNA dynamics were not primarily caused by reduced neutrophil viability.

Flow cytometric analysis of Annexin V/Propidium Iodide (PI) stained co-cultures revealed polarization-dependent differences in the phenotypes associated with neutrophil cell death (**Figure 5F**). IFN-γ/LPS-polarizing conditions were associated with an increased frequency of PI and Annexin V double-positive neutrophils, suggesting an increased loss of membrane integrity, particularly in concurrently polarized co-cultures. In contrast, IL-4/IL-13-polarizing conditions were associated with a relative increase in Annexin V^+^/PI^–^ neutrophils, consistent with an early apoptotic phenotype.

### 3.5 Macrophages polarization environment shapes the cytokine response to macrophage-neutrophil co-culture

**Figure 6:**
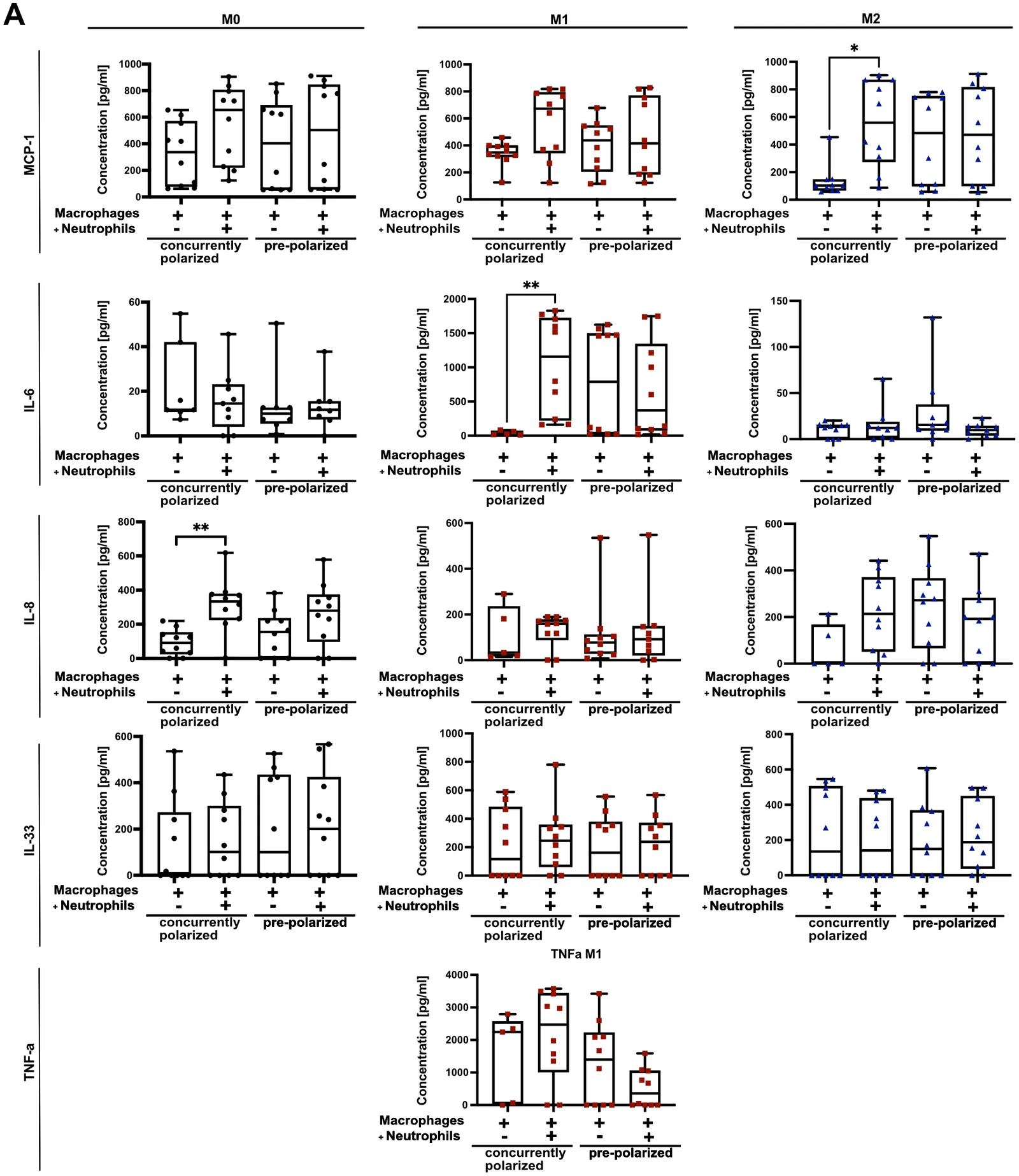
Macrophage polarization environment shapes cytokine and chemokine responses to neutrophils co-culture. Cytokine and chemokine concentrations (MCP-1, IL-6, IL-8, IL-33, TNF-α) in supernatants of M0, M1, and M2 macrophages cultured alone or with neutrophils, quantified by LEGENDplex™ multiplex immunoassay. Concurrently polarized and pre-polarized conditions are shown. *p < 0.05, **p < 0.01 (Kruskal–Wallis test followed by Dunn’s multiple comparisons test for comparisons of more than two groups). n = 10 independent donors.

To assess how neutrophils modulate macrophage inflammatory responses, cytokine and chemokine concentrations were quantified in culture supernatants of M0, M1, and M2 macrophages cultured alone or with neutrophils by multiplex immunoassay (**Figure 6**).

In M0 cultures, neutrophil co-culture induced selective modulation of inflammatory mediators, including changes in MCP-1, IL-6, and IL-8 secretion, compared with macrophage monocultures. The most prominent neutrophil-dependent increase was observed for IL-8 in concurrently polarized macrophage cultures, whereas responses were less pronounced after macrophage pre-polarization. These findings indicate that cytokine responses differ according to the timing and length of exposure to polarizing stimuli.

Under IFN-γ/LPS (M1-polarized) conditions, macrophage–neutrophil co-culture was associated with increased inflammatory mediator concentrations. IL-6 concentrations were significantly increased in concurrently polarized M1 macrophage–neutrophil co-cultures, whereas this response was reduced after macrophage pre-polarization. TNF-α secretion followed a similar trend, with higher concentrations under concurrently polarized inflammatory conditions and reduced levels after macrophage pre-polarization in the presence of neutrophils. MCP-1 and IL-8 secretion showed additional neutrophil-associated increases, although responses varied between individual donors. Together, these findings indicate that neutrophils amplify inflammatory cytokine production most efficiently in macrophages undergoing active polarization.

In contrast, IL-4/IL-13 (M2-polarized) cultures displayed a distinct cytokine profile. Despite limited induction of classical inflammatory cytokines such as IL-6, neutrophil co-culture increased selected chemokines, particularly MCP-1 under concurrently polarized IL-4/IL-13 conditions. IL-8 responses were detectable but showed considerable donor-dependent variability, whereas IL-33 concentrations were not consistently altered by neutrophil addition across M0, IFN-γ/LPS, or IL-4/IL-13 conditions. Together with preserved neutrophil populations and reduced lytic cell death (see **Figure 5**), these cytokine patterns support regulated neutrophil–macrophage crosstalk rather than a response dominated by unspecific inflammatory cell damage.

Neutrophil-only controls, stimulated under the same conditions as concurrently polarized co-cultures, are shown in **Supplementary Figure 6**. Here, only MCP-1 and IL-8 were measurable cytokines. This suggests that the elevated concentrations of inflammatory mediators are more likely due to a reaction associated with the co-culture than to secretion by neutrophils alone in response to the polarization stimuli.

### 3.6 Soluble mediators are sufficient to regulate macrophage polarization and neutrophil NETs-associated extracellular DNA formation in a transwell co-culture system

**Figure 7:**
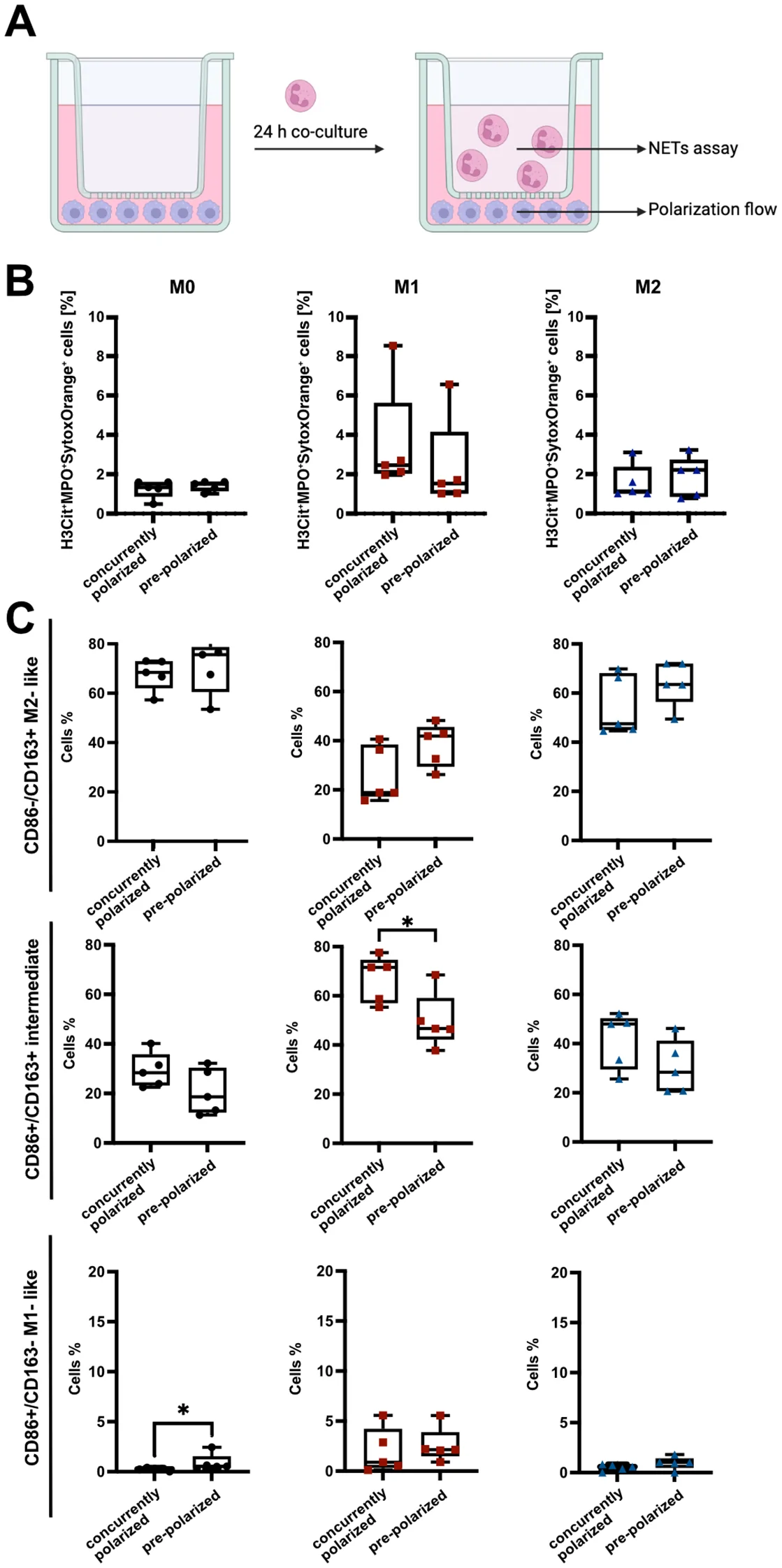
Soluble mediators modulate macrophage polarization and induce NETs formation in a transwell co-culture system. **(A)** Schematic overview of the transwell co-culture model. Macrophages were cultured in the lower chamber and neutrophils were added to the upper insert, allowing exchange of soluble mediators while preventing direct cell–cell contact. After 24 h of co-culture, NETs release and macrophage polarization were analyzed by flow cytometry. **(B)** Quantification of H3cit^+^MPO^+^Sytox™ Orange^+^ events as markers of NETs in concurrently polarized (CP) and pre-polarized (PP) M0, M1, and M2 macrophage–neutrophil transwell co-cultures. **(C)** Flow cytometric analysis of macrophage polarization states after transwell co-culture with neutrophils. Macrophage subsets were defined as CD86^–^/CD163^+^ M2-like, CD86^+^/CD163^+^ intermediate, and CD86^+^/CD163^–^ M1-like populations and quantified in M0, IFN-γ/LPS and IL-4/IL-13 conditions comparing concurrently polarized and pre-polarized macrophages. *P < 0.05 (Mann–Whitney U test for comparisons between two groups). n = 5 independent donors.

To determine whether neutrophil-mediated macrophage modulation requires direct cell–cell contact or is mediated by soluble factors, macrophages and autologous neutrophils were cultured in a transwell system that allows exchange of soluble mediators while preventing direct cellular interaction (**Figure 7A**). Concurrently polarized and pre-polarized M0, M1, and M2 macrophages were cultured in the lower compartment, while neutrophils were added to the transwell insert for 24 h. Subsequently, NETs-associated extracellular DNA formation was assessed in neutrophils, and macrophage polarization profiles were analyzed separately by flow cytometry.

Flow cytometric detection of H3Cit^+^MPO^+^SytoxOrange^+^ neutrophils demonstrated that macrophage-derived soluble mediators influenced NETs formation even in the absence of direct cell contact (**Figure 7B**). H3Cit^+^MPO^+^SytoxOrange^+^ events were detectable across all macrophage polarization conditions, indicating that soluble signals exchanged between macrophages and neutrophils are sufficient to induce NETs-associated responses. Differences between concurrently polarized and pre-polarized macrophage conditions remained modest. It is remarkable that the frequency of H3Cit^+^MPO^+^SytoxOrange^+^ events was higher in the Transwell system compared to direct co-culture experiments, suggesting that soluble factors derived from macrophages are sufficient to induce NETs-associated chromatin responses, and that direct contact between macrophages and neutrophils is not required for NETs induction under these conditions.

Under IFN-γ/LPS (M1-polarized) conditions, pre-polarization significantly reduced the proportion of CD86^+^CD163^+^ intermediate macrophages, indicating that stimulation length influences macrophage responses to soluble neutrophil-derived mediators. Under IL-4/IL-13 (M2-polarized) conditions, macrophages maintained dominant CD163-associated profiles regardless of polarization timing.

Taken together, these results show that soluble mediators are sufficient to support bidirectional communication between macrophages and neutrophils. This leads to altered NETs formation, altered macrophage polarization, and altered extent of NETs-associated reactions compared with direct co-culture.

## 4 Discussion

In this study, we systematically investigated the bidirectional regulation between macrophage polarization and NETs dynamics using an autologous human primary cell co-culture system. Our findings reveal that macrophage activation state coordinately determines NETs formation, NETs-associated chromatin accumulation, extracellular DNA persistence, neutrophil cell death pathways, and inflammatory cytokine output, and that neutrophils reciprocally reprogram macrophage activation state, with the magnitude of this effect critically dependent on the degree of prior macrophage polarization and the polarizing environment.

A key observation was the pronounced phenotypic heterogeneity of monocyte-derived macrophages, even under defined polarizing conditions. Intermediate CD14^+^CD16^+^ monocytes predominated in early cultures and progressively transitioned to CD68^+^ macrophages, consistent with established maturation trajectories. Importantly, polarization markers including CD86, CD80, CD206, and CD163 were detectable in monocytes before full macrophage differentiation, suggesting that functional polarization is initiated prior to phenotypic maturation (23). The persistence of mixed CD86^+^CD163^+^ populations under IFN-γ/LPS or IL-4/IL-13 conditions reinforces the concept that macrophage activation constitutes a continuum rather than a binary switch (22). Importantly, the use of M-CSF for macrophage differentiation may influence the baseline activation state of macrophages. M-CSF-derived macrophages have been reported to display tissue-resident and M2-associated characteristics, which may affect their subsequent response to inflammatory stimulation. Therefore, M1 polarization in this study should be interpreted as functional inflammatory reprogramming of M-CSF-derived macrophages rather than generation of a completely distinct macrophage phenotype.

The observation that neutrophils consistently promoted CD163-associated features across all conditions extends previous reports that efferocytosis of apoptotic neutrophils drives M2 polarization (24, 25). Critically, this effect was most pronounced in concurrently polarized macrophages and attenuated in pre-polarized cultures, suggesting that the timing and duration of exposure to polarization stimuli influence the macrophages’ response to co-culture with neutrophils. The persistent expression of CD86 in M2-shifted co-cultures may reflect an M2b-like activation state characterized by retention of costimulatory molecules alongside regulatory functions, rather than incomplete polarization (26–29).

A major challenge in NETs biology is distinguishing regulated NETs formation from extracellular DNA release caused by necrosis, late apoptosis, or other forms of lytic cell death. Since their initial description by Brinkmann et al. (4), NETs have been recognized as extracellular chromatin structures equipped with antimicrobial proteins such as NE and MPO, which contribute to the host’s immune response but can also promote inflammatory tissue damage when dysregulated (7). Mechanistically, NETs formation involves chromatin remodeling mediated by NE and MPO, as well as PAD4-dependent histone citrullination (30).

Therefore, we expanded our analysis beyond Sytox-based extracellular DNA measurements and combined complementary approaches including H3Cit/MPO/SytoxOrange flow cytometry, MPO-DNA and NE-DNA complex detection, IF staining, live-cell imaging, PAD4 inhibition using GSK-484, and neutrophil cell death analysis.

Together, these complementary approaches strengthen the identification of NETs-associated chromatin responses while acknowledging that no single assay alone specifically defines NETs formation and that reliable assessment of NETs formation requires combined analysis of extracellular DNA release, NETs-associated proteins, chromatin modifications, and cellular morphology (4, 7, 31).

Across all NETs-associated measurements, an inflammatory FN-γ/LPS (M1-polarized) environment consistently promoted increased accumulation of NETs (32, 33). Pre-polarized macrophage cultures showed reduced neutrophil-associated NETs responses compared with concurrently polarized counterparts, consistent with an influence of the timing and duration of exposure to polarizing stimuli on subsequent co-culture responses (21, 32). IL-4/IL-13 (M2-polarized) environments showed significantly reduced NETs DNA accumulation and NE activity suggesting active restriction of NETs dynamics rather than complete suppression (34).

The detection of H3Cit^+^MPO^+^SytoxOrange^+^ events provided additional evidence for NETs, as PAD4-mediated histone citrullination contributes to chromatin decondensation during NETs formation (6).

These findings complement the SytoxGreen-based kinetic assays, which primarily reflect extracellular DNA accumulation and cannot independently distinguish NET formation from other sources of DNA release.

Representative live-cell imaging provided additional qualitative insight into the temporal interaction between macrophages and neutrophil-derived extracellular DNA structures. Although this approach is not intended as a quantitative readout, it illustrates dynamic processes, including extracellular DNA release, neutrophil–macrophage interactions, and subsequent processing of extracellular material over time.

Pharmacological inhibition of PAD4 using GSK-484 altered extracellular DNA kinetics, supporting a contribution of PAD4-dependent chromatin decondensation to the observed extracellular DNA response. However, PAD4 inhibition did not completely abolish SytoxGreen signals, indicating that extracellular DNA accumulation reflects a combination of PAD4-dependent NETs formation, PAD4-independent DNA release, additional neutrophil death pathways, and differences in extracellular DNA persistence (5, 35).

The time-resolved analysis revealed state-dependent differences in the persistence of extracellular DNA. However, the present experiments do not directly distinguish between reduced DNA release and enhanced degradation or increased uptake. Nakazawa et al. demonstrated that macrophages actively interact with NETotic neutrophils and contribute to the clearance of NETs-derived material, supporting an important role of macrophages in regulating extracellular chromatin persistence and resolution of neutrophil responses (20). The higher phagocytic capacity of M2 macrophages described by Suleimanov et al. (36) may contribute to differences in extracellular DNA persistence under IL-4/IL-13-polarizing conditions.

To further determine whether macrophage-mediated regulation of NETs responses requires direct cellular interaction or can occur through soluble mediators, we performed transwell experiments that separated macrophages and neutrophils while allowing the exchange of soluble factors. These experiments demonstrated that soluble macrophage- and neutrophil-derived signals were sufficient to regulate both NETs formation responses and macrophage polarization-associated marker expression.

The increased accumulation of H3Cit^+^MPO^+^SytoxOrange^+^ events after physical separation suggests that soluble mediators are sufficient to modulate NETs-associated chromatin release, whereas direct interaction may facilitate macrophage-mediated processing and clearance of extracellular NETs material. These findings are supported by Manda-Handzlik et al., who demonstrated that macrophage-derived secretomes regulate NETs release independently of direct cellular contact (7). Nevertheless, this study provides no insight into whether the differences between direct and Transwell co-culture are attributable to altered NETs formation, the persistence of extracellular DNA, or macrophage-mediated processing.

Analysis of neutrophil cell death by Annexin V/PI staining revealed polarization-dependent differences. Under IFN-γ/LPS conditions (M1-polarized), there was an increase in both the PI-positive and Annexin V^+^/PI^+^ populations, suggesting an increased loss of membrane integrity. In contrast, IL-4/IL-13 (M2-polarized) conditions were associated with a relative increase in Annexin V^+^/PI^–^ neutrophils and fewer PI-positive cells. This profile favors efferocytic clearance over secondary lysis and limits the release of damage-associated molecular patterns (37–39). However, Annexin V/PI staining alone cannot uniquely define the underlying mode of cell death. Importantly, quantification of Hoechst-positive neutrophil populations after 24 h showed that neutrophils persisted throughout the experimental period, indicating that differences in extracellular DNA signals were not solely due to loss of neutrophil populations. Together with Annexin V/PI analysis, these findings suggest that macrophage polarization influences the balance between regulated neutrophil responses and lytic cell death pathways.

The cytokine profiles mirrored these functional differences: neutrophils amplified IL-6 and TNF-α release in inflammatory macrophage environments (40, 41), and IL-4/IL-13-stimulated cultures showed attenuated inflammatory cytokine profiles (32, 42). The stability of IL-33 across conditions argues against non-specific alarmin release and supports regulated intercellular signaling (43, 44).

Several limitations of this study warrant consideration. First, our findings are based on an *in vitro* co-culture system that cannot fully recapitulate the complexity of *in vivo* tissue microenvironments including the contributions of other immune and stromal cell types, extracellular matrix components, and spatial organization. Second, the use of M-CSF for monocyte-to-macrophage differentiation may influence the baseline activation state of macrophages, as M-CSF-derived macrophages have been described to exhibit a more tissue-resident and M2-like phenotype (45). This pre-existing differentiation bias may affect the extent of subsequent inflammatory activation, and therefore M1 polarization should be interpreted as functional reprogramming of M-CSF-derived macrophages rather than the generation of a completely distinct macrophage phenotype. Third, our analysis relied on surface marker expression and functional readouts without transcriptomic or epigenetic profiling. Single-cell RNA sequencing would provide deeper insight into the heterogeneity of macrophage activation states and their transcriptional regulation during co-culture. Fourth, we did not assess the functional consequences of altered NETs dynamics for antimicrobial defense or tissue injury which would require pathogen challenge or *in vivo* models. Future studies incorporating intravital imaging, conditional knockout models, and patient-derived cells from inflammatory conditions could address these limitations and establish the *in vivo* relevance of the mechanisms identified here. Fifth, although our revised experimental approach combines multiple complementary NETs-associated readouts, definitive discrimination between NETs formation and other extracellular DNA sources remains challenging. Sytox-based measurements and MPO-DNA detection alone cannot exclusively identify NETs formation; therefore, our conclusions rely on combined evidence from chromatin modification (H3Cit), granule protein association (MPO/NE), PAD4 inhibition, imaging approaches, and neutrophil cell death profiling. Furthermore, NET degradation was not measured directly. A decrease in SYTOX fluorescence may indicate reduced DNA release, reduced degradation, or reduced cellular uptake; therefore, our kinetic data and the live-cell imaging data from a single donor are interpreted as a measure of the dynamics of extracellular DNA and not as evidence of enhanced NETs degradation. Furthermore, since polarizing stimuli were still present during the co-culture, the observed responses reflect the combined influence of the polarizing environment and macrophage-neutrophil interactions and cannot be attributed exclusively to macrophage-intrinsic polarization states. The polarizing agents were not removed at any point: macrophages were not washed and were not transferred to cytokine-free medium before neutrophil addition. Consequently, neutrophils were directly exposed to IFN-γ/LPS or IL-4/IL-13 for 24 h in both the concurrently polarized (CP) and pre-polarized (PP) condition, whereas cumulative macrophage exposure differed (24 h in CP versus 48 h in PP).

In summary, our data demonstrate that macrophage–neutrophil interactions are governed by a dynamic continuum of macrophage activation states rather than a rigid M1/M2 dichotomy. Concurrently polarized macrophages are particularly susceptible to neutrophil-mediated reprogramming, whereas macrophages with a longer prior exposure to polarizing stimuli show more limited secondary modulation. The polarizing environment and macrophage–neutrophil interactions shape NETs-associated extracellular DNA dynamics and neutrophil survival pathways. These findings identify the polarizing environment and stimulation duration as important determinants of bidirectional macrophage–neutrophil responses.

## Data Availability

The raw data supporting the conclusions of this article will be made available by the authors, without undue reservation.
