# Supplementary material for: Macrophage polarization modulates neutrophil extracellular trap dynamics and reciprocal macrophage responses
Source: Front Immunol. 2026 Sep 1;17:1850388. doi: 10.3389/fimmu.2026.1850388 (PMC13574648; doi:10.3389/fimmu.2026.1850388)
Supplement: Supplementary file 1 [file DataSheet1.pdf]

frontiers

The International Society for the Advancement of Cytometry (ISAC) has highlighted the importance of including comprehensive methodological information to ensure data reproducibility and reliability. In line with this, Frontiers in Immunology now requires authors to submit a checklist for manuscripts that involve flow or mass cytometry. This checklist helps standardize the reporting process, improving the quality and transparency of published data. By doing so, we support scientific progress, making it easier for other researchers to replicate and validate experiments.

This form should be submitted with any manuscripts using flow or mass cytometry.

Sample/specimen/material description

☐ Total blood

☑ PBMCs

☐ Organ digests

Other PMN

Did the samples suffer any treatment before or after incubation with the antibodies?

☑ Drug GSK-484

☐ Cell permeabilization ______________________

☑ Dye CFSE, FAR Red

☑ Propidium iodine

☐ Not applicable

Other ______________________________________

Instrument and antibodies

Name of the Cytometer Attune CytPix

**Antibodies and targets**	**Fluorochrome/ Metal**	**Catalog number/Company**
e.g. anti-CD4	FITC	Cat. XXX/ XXX ltd.
CD14	Brilliant Violet 605™	301834/BioLegend

CD68	PE	12-0689-42/Thermo Fisher Scientific
CD86	NovaRed	H021T03R06-A/Thermo Fisher Scientific
CD163	PE/Cyanine7	333614/BioLegend
CD16	Pacific Blue	558122/BD
Annexin V	FITC	31490013X2/Immuno Tools
MPO	eFluor 450	48-1299-42/Thermo Fisher Scientific
SytoxOrange		S34861/Thermo Fisher Scientific
Hoechst 33342		62249/Thermo Fisher Scientific
Goat anti-Rabbit IgG (H+L) Cross-Adsorbed	Alexa Fluor™ 700	A-21038/Thermo Fisher Scientific

Data analyses

1. Name of the software FlowJo 10 _______________________________
2. Reference gating strategy in the manuscript or supplementary material

Gating strategy in (eg Figure X) Supplement 1-4 ______________________________
