# Supplementary material for: Macrophage polarization modulates neutrophil extracellular trap dynamics and reciprocal macrophage responses
Source: Front Immunol. 2026 Sep 1;17:1850388. doi: 10.3389/fimmu.2026.1850388 (PMC13574648; doi:10.3389/fimmu.2026.1850388)
Supplement: Supplementary file 2 [file Table1.docx]

**Supplement**

**Figure 1: Gating strategy for macrophage polarization.** Co-culture was gated on CSFE-stained macrophages and CD68+ macrophages. To identify phenotype, CD68+ macrophages were gated on CD86/CD163.

**Figure 2: Gating strategy for Annexin/PI/Hoechst.**

**Figure 3: Gating strategy for MPO/SytoxOrange staining.**

**Figure 4: Gating strategy for H3cit/MPO/SytoxOrange staining**

**Figure 5: Representative immunofluorescence images illustrating NETs-associated extracellular DNA morphology (A)** Representative immunofluorescence images of macrophages and neutrophils cultured under M1 and M2 conditions stained for DAPI (blue), Sytox Green (extracellular and nuclear DNA, green), MPO (red), and merged images. 20x magnification, scale bars: 100 µm. **(B)** DNA-focused images showing only DAPI (blue) and Sytox Green (green) to facilitate visualization of extracellular DNA morphology independent of MPO staining. 20x magnification, scale bars: 100 µm. (**C)** Higher-magnification representative images of DAPI (blue) and Sytox Green (green) highlighting extracellular DNA structures.60x magnification, scale bars: 50 µm. NOTE: Sytox Green was applied after fixation and therefore stains DNA in fixed cells (nucleus) as well as extracellular DNA. Intracellular Sytox Green signal largely overlaps with DAPI-positive nuclei, while Sytox Green-positive signals extending beyond DAPI-defined nuclei are consistent with extracellular DNA-associated structures.

**Figure 6: Neutrophils only immune profile.** Cytokine and chemokine concentrations (MCP-1, IL-8) in supernatants of neutrophils cultured with M-CSF (M0 environment), IFN-γ/LPS (M1 environment) or IL-4/IL-13 (M2 environment), quantified by LEGENDplex™ multiplex immunoassay. n = 5 independent donors.
